# Neurobiological Basis of Increased Risk for Suicidal Behaviour

**DOI:** 10.3390/cells10102519

**Published:** 2021-09-23

**Authors:** Aleksandra Wisłowska-Stanek, Karolina Kołosowska, Piotr Maciejak

**Affiliations:** 1 Centre for Preclinical Research and Technology (CEPT), Department of Experimental and Clinical Pharmacology, Medical University of Warsaw, 1B Banacha Street, 02-097 Warsaw, Poland; pmaciejak@ipin.edu.pl; 2 Department of Neurochemistry, Institute of Psychiatry and Neurology, 9 Sobieskiego Street, 02-957 Warsaw, Poland; kkolosowska@ipin.edu.pl

**Keywords:** suicide, serotonin, kynurenine pathway, cortisol, BDNF, epigenetics, lithium, ketamine, esketamine, clozapine

## Abstract

According to the World Health Organization (WHO), more than 700,000 people die per year due to suicide. Suicide risk factors include a previous suicide attempt and psychiatric disorders. The highest mortality rate in suicide worldwide is due to depression. Current evidence suggests that suicide etiopathogenesis is associated with neuroinflammation that activates the kynurenine pathway and causes subsequent serotonin depletion and stimulation of glutamate neurotransmission. These changes are accompanied by decreased BDNF (brain-derived neurotrophic factor) levels in the brain, which is often linked to impaired neuroplasticity and cognitive deficits. Most suicidal patients have a hyperactive hypothalamus–pituitary–adrenal (HPA) axis. Epigenetic mechanisms control the above-mentioned neurobiological changes associated with suicidal behaviour. Suicide risk could be attenuated by appropriate psychological treatment, electroconvulsive treatment, and drugs: lithium, ketamine, esketamine, clozapine. In this review, we present the etiopathogenesis of suicide behaviour and explore the mechanisms of action of anti-suicidal treatments, pinpointing similarities among them.

## 1. The Scope of the Problem

According to the World Health Organization (WHO), more than 700,000 people die each year due to suicide, while suicide attempts are 10–20 times more frequent [1]. Suicide accounts for 8.5% of deaths among adolescents and young adults (15–29 years) and is the second leading cause of death among this population worldwide. Completed suicides are three times more common in males than females, while for suicide attempts, an inverse ratio is observed [2]. Suicide risk factors include a previous suicide attempt, non-suicidal self-harm, and psychiatric disorders [3,4,5]. It is estimated that 90% of suicide victims have at least one mental disorder [3,4,6]. Over 50% of suicides occur in patients with major depression or bipolar disorders, especially when they are treatment resistant [3,7,8]. On the one hand, thirty percent of patients with treatment-resistant depression attempt suicide at least once during their life [9]. The second cause of suicide is alcohol abuse [10]. Other risk factors are schizophrenia, post-traumatic stress disorder (PTSD), anorexia nervosa, sleep disorder, antisocial personality disorder, borderline personality disorder, and/or substance abuse [3,7,11,12]. On the other hand, only 5% of psychiatric patients commit suicide. This particular risk group includes patients requiring hospital treatment (10 times higher than in the general population) [13]. In addition to psychiatric conditions, suicidal behaviour has been associated with chronic diseases, especially those with unfavourable prognoses or accompanying chronic pain such as HIV infection or cancer [2,14,15].

Traits that increase the probability of suicidal behaviour include aggression, impulsivity, pessimism, hopelessness, impaired cognitive functions, and poor affect regulation [3,16,17]. Higher levels of impulsivity and aggression are especially seen in younger suicide victims [18].

Suicidal behaviour is thought to be triggered by an interplay between genetic, psychological, and environmental factors [19]. The estimated heritability of suicidal behaviour has been shown to range from 30 to 55% in twin studies [19,20]. Several studies have suggested that the likelihood of attempting or committing suicide is ten times higher in relatives of suicide completers. Genome-wide association studies (GWAS) have indicated that polygenic risk and specific loci such as genes involved in circadian clock regulation, tyrosine metabolism, and risk factors for depressive disorder are associated with a higher risk of attempting suicide [20,21]. The stress-diathesis model assumes that stressors interact with neurobiological and psychological susceptibilities to cause suicidal behaviour as the maladaptive stress response [22,23,24,25,26]. A chronic and acute stressful situation such as deprivation, isolation, family adversity, sexual abuse, school, employment and financial difficulties, and experiences of loss and death can increase the incidence of suicidal behaviour [27,28]. Data suggest that early life adversity is especially harmful and increases the rate of impulsive and suicidal behaviour by two to five times [6,29,30]. Table 1 summarises the risk factors and factors associated with a decreased risk of suicide.

## 2. The Biological Background of Suicidal Behaviour

Many psychological tools can be useful to evaluate the risk of suicidal behaviour, such as the Suicide Intent Scale, the Suicide Assessment Scale, the Karolinska Interpersonal Violence Scale, the Columbia Suicide Severity Rating Scale, the Beck Hopelessness Scale, and the Harcavy-Asnis Suicide Scale [15,31,32]. Some scales help to validate the risk of suicide in children and/or adolescents, such as the Child Suicide Potential Scale, the Evaluation of Suicide Risk Among Adolescents, and Imminent Danger Assessment [33,34]. All these scales are based on asking individuals about suicidal intentions and risk factors of suicide. However, in many cases, suicide scales provide insufficient predictive validity for future suicide attempts. They are limited by a patient’s willingness to share information, as well as a high rate of false positives. To date, the best method to assess a patient’s risk for suicide is screening for past suicide attempts. Hence, searching for potential biological markers could be very beneficial, especially in situations associated with an increased risk of suicide, including a severe episode of depression, psychosis, or hospitalisation.

Suicide etiopathogenesis seems to be associated with neuroinflammation that stimulates the kynurenine pathway and causes the depletion of serotonin and melatonin [31]. A decreased level of serotonin is associated with aggression and impulsivity [35]. Proinflammatory cytokines activate the hypothalamic–pituitary–adrenal axis [36]. Moreover, increased metabolism of kynurenic acid into neurotoxic quinolinic acid, an NMDA receptor agonist, results in glutamatergic system overactivation and a decrease in the production of BDNF, which worsens neuroplasticity and could cause cognitive problems [37].

**Figure 1:**
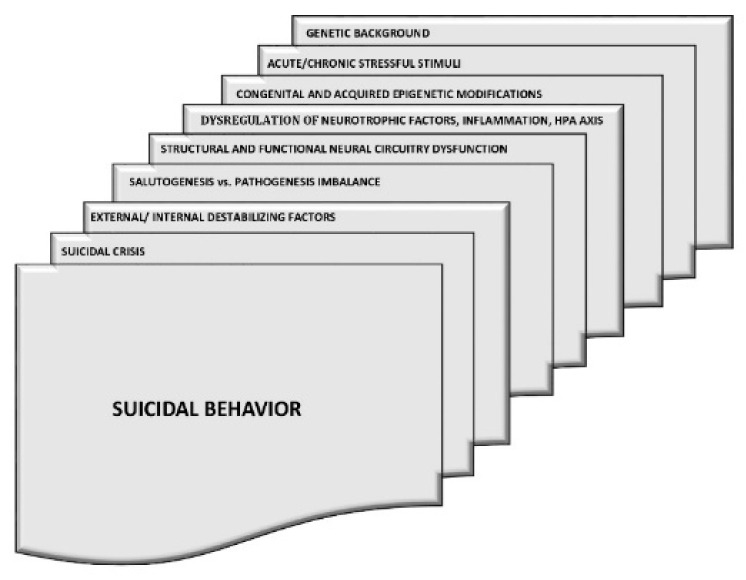
Biological factors that are likely to lead to suicidal behaviour.

The Niculescu group suggested the following potential markers of suicidality: interleukin-6 (IL-6), MAO-B (monoamine oxidase-B), apolipoprotein E (involved in fat metabolism), and SAT1 (spermidine/spermine N1-acetyltransferase 1) [38,39]. SAT1 is a key catabolic enzyme for polyamines. Polyamine levels within cells control cell viability, while significant decreases in polyamine levels can result in apoptosis [40]. SAT1 is highly inducible by stress and cytokines [40]. In men reporting suicidal ideation, a high level of tumour necrosis factor (TNFSF10, ligand superfamily, Member 10) was observed in the blood [40]. For bipolar disorder, SLC4A4 (which regulates brain pH) predicted suicidal ideation and future hospitalisation [38]. Numerous studies have identified insomnia and altered sleep architecture as predictors of suicidal thoughts and behaviour [41,42,43]. Figure 1 and Table 2 summarise the biological factors associated with suicidal behaviours.

### 2.1. Inflammation and the Kynurenine Pathway

Inflammatory mediators play a critical role in the pathophysiology of suicide [1,30,44]. Suicidal patients display elevated markers of inflammation in the central nervous system and peripheral tissues, irrespective of their primary diagnosis, age, and gender [1,45].

A prospective study of 300,000 women (1000 of them later died due to suicide) revealed that a higher level of white cells was predictive of suicide [46]. CRP (C-reactive protein) acute-phase protein level in plasma has been correlated with suicidal intent, but not the number of attempts or severity of violence [25,46,47]. Inflammatory cytokines such as IL-1 beta and IL-6 have been shown to be increased in the blood and CSF of suicidal patients [44,48,49]. Simultaneously, decreased neuroprotective IL-8 has been observed in the plasma and CSF of suicidal patients [46,49]. The messenger ribonucleic acid (mRNA) and protein levels of IL-1β, IL-6, and TNF-α were abnormally elevated in the prefrontal cortex of young adults who died by suicide [49]. An elevated level of TNF-α was noticed in the dorsolateral prefrontal cortex of individuals who died by suicide, regardless of psychiatric diagnosis [23]; this observation supports the fact that interferon treatment increases the risk of depression and suicide [50,51]. It is worth mentioning that aberrant levels of proinflammatory cytokines are not specific to suicide but are also reported in major depressive disorder, bipolar disorder, and schizophrenia [52,53].

The importance of inflammation in suicide and depressive behaviour is also supported by observations and trials with nonsteroidal anti-inflammatory drugs (NSAIDs). Reports have suggested significantly less suicidal ideation in patients treated with ibuprofen, naproxen, celecoxib, or aspirin as compared with acetaminophen (paracetamol) [54]. Moreover, a meta-analysis of 36 randomised trials found that NSAID augmentation in patients with major depression improved antidepressants’ treatment effects [55]. Most data suggest enhanced treatment response for celecoxib [56,57]. The effect is particularly evident in patients with elevated inflammation markers such as CRP [56]. Otherwise, it is also worth remembering that over the counter (OTC) NSAIDs are responsible for about 50% of suicidal attempts [58].

Another vital link between inflammation and suicidal behaviour is tryptophan metabolism via the kynurenine pathway [31]. The kynurenine pathway is responsible for over 90% degradation of tryptophan in the periphery and is present in many tissues such as the brain, liver, intestine, and immune cells [1]. The first step of the kynurenine pathway is converting tryptophan to N-formylkynurenine by enzyme indoleamine-2,3-dioxygenase (IDO) or tryptophan 2,3 dioxygenase (TDO). In the following steps, kynurenine, quinolinic acid (QUIN), kynurenic acid (KYNA), and picolinic acid (PIC) are produced [1]. One of the final products of kynurenine is NAD^+^ [59]. Proinflammatory cytokines such as IFN-γ, IL-1β, and IL-6 activate the kynurenine pathway via stimulation of the IDO, which results in increased synthesis of quinolinic acid (QUIN) and/or kynurenic acid, and simultaneous depletion of serotonin and melatonin; therefore, QUIN levels could achieve neurotoxic level [31,48,59,60]. QUIN is a potent excitotoxin with NMDA agonistic activity [48]. The increased QUIN level produced by activated microglia might contribute to neuronal loss and reduced hippocampal volume [31]. QUIN can interact with free iron ions to form toxic complexes that exacerbate oxygen species radical formation, oxidative stress, and mitochondrial dysfunction [59]. In the CSF of suicide attempters and patients with suicidal intent, increased levels of QUIN were observed, regardless of mood disorder comorbidity [31,61]. The increase of QUIN in CSF patients who have recently attempted suicide was very potent, i.e., about 300% [31]. There was a significant correlation among CFS levels of QUIN acid, IL-6, and the suicidal ideation scores on the Suicide Intent Scale [31,47].

Moreover, as compared with nonviolent attempters, violent attempters have higher QUIN levels in their CSF [31]. Patients with suicide attempts and depression have higher kynurenine levels than patients with depression without suicide attempts [1,31,62]. Another metabolite of the kynurenic pathway is neuroprotective picolinic acid (PIC); its level was reduced in the CSF and blood of suicide attempters [48]. Similarly, suicide attempters have been characterised by a decreased PIC/QUIN ratio in their CSF and blood [48]. The reduction of PIC in the CSF was sustained over two years after a suicide attempt [48]. Further, anti-suicidal procedures, such as electroconvulsive therapy or ketamine can alter kynurenine metabolism, which suggest that this metabolic pathway may be helpful as a monitoring marker [48,63].

### 2.2. Serotonin System

Serotonin deficits are implicated in pathogenesis of depression and also in aggression, impulsivity, suicidal ideations, and suicide attempts [3,35,64,65,66]. The CSF’s lower levels of the major serotonin metabolite 5-hydroxyindoleacetic acid (5-HIAA) were found in suicide attempters with psychiatric disorders as compared with psychiatric non-attempters and healthy controls [3,35,65,66]. Moreover, lower levels of 5-HIAA in the CSF corresponded with the lethality of suicide attempt and predicted future suicide attempts and completion [3,67]. Lower 5-HIAA levels were also observed in platelets of suicidal patients [35]. Most studies of suicide victims have reported a decreased density of serotonin transporter in the prefrontal cortex, anterior cingulate, and hypothalamus, but some studies have shown no changes [3,68,69]. Most studies have reported that suicidal patients had upregulated expression of 5HT_1A_ and 5HT_2A_ receptors in the raphe and the prefrontal cortex, probably as a compensatory response to low activity of serotoninergic neurons. However, some studies have found no differences or even decreased 5HT_1A_ and 5HT_2A_ receptors’ expression. Moreover, increased expression of serotoninergic receptors has been observed in platelets [3,30,35,70,71]. This hypothesis suggests that depleted serotonin levels result from enhanced tryptophan metabolism to QUIN via the kynurenine pathway. Serotonin deficiency may decrease neurogenesis and lead to cognitive deficits. Moreover, studies have reported that polymorphisms of enzymes associated with serotonin synthesis and metabolism, such as tryptophan hydroxylase (polymorphism of intron 7) and MAO-A, were connected to suicide [72].

### 2.3. Brain-Derived Neurotrophic Factor (BDNF)

Suicidal patients are characterised by abnormal neuroplasticity [23,73]. Data indicate lower mRNA levels of BDNF and its receptor TrkB in the prefrontal cortex and hippocampus in suicide patients [74,75,76]. Moreover, downregulation of BDNF in the anterior cingulate cortex and amygdala, and lower serum BDNF levels, have been reported in depressed patients with suicidal intentions as compared with depressed patients without suicidal intentions [30,73,75]. Lower levels of BDNF in suicidal patients are probably the consequence of epigenetic modifications due to stress [75,76]. Some data suggest that serum levels of BDNF could be a promising marker of suicide [76], but others deny this finding [73].

### 2.4. The Hypothalamic–Pituitary–Adrenal (HPA) Axis

Suicidal behaviours seem to be associated with hyperactivity of the HPA axis, which may cause disturbed control of stress, impaired function of the hippocampus, and cognitive deficits [3]. Several studies have revealed that non-suppressors in dexamethasone test are more likely to commit suicide [35,77,78,79]. Adrenal gland cortical hypertrophy was highlighted in patients who died by suicide [80]. Higher cortisol levels in saliva, CSF, and plasma have been reported in suicide attempters than in healthy volunteers [79,81]. Depressed suicide patients had increased corticotrophin-releasing hormone (CRH) levels in the paraventricular nucleus of the hypothalamus, forebrain, locus coeruleus, and fewer CRH_1_ receptors in the frontopolar cortex [70,81,82] (Jokinen et al., 2018; Merali et al., 2004; Oquendo et al., 2014). Protein and gene expression of GR-α (glucocorticoid receptor) were significantly decreased in the prefrontal cortex and amygdala of teenage suicide victims as compared with controls [69,83].

### 2.5. Glutaminergic and GABAergic Neurotransmission 

The effectiveness of the glutaminergic NMDA receptor antagonists, ketamine/esketamine, in decreasing suicide rates suggests the involvement of glutamate in this process. Existing data demonstrate that glutamate may play an important role in suicide-related personality traits, including impulsivity and aggression [17]. This phenomenon could be associated with increased levels of the NMDA receptor agonist QUIN in the central nervous system of suicide attempters due to activation of the kynurenine pathway as a consequence of stimulation of IDO by proinflammatory cytokines [31]. Subsequently, most studies have reported decreases or no difference in NMDA binding in the prefrontal cortex in cases of suicide [70,84,85].

Data suggest a disturbed balance between glutaminergic and GABAergic neurotransmission in suicide risk. Nevertheless, there are some discrepancies in the research. Suicide victims are characterised by an increased expression of GABA-A receptors in the hippocampus and prefrontal cortex [86,87]. In addition, a pilot study examining the GABAergic system in suicide found decreased GABA-A gamma subunit expression in the prefrontal cortex of patients with depressive disorder and schizophrenia [88]. Data from a small group (12 suicide and 12 control participants) revealed decreased expression of α1, α3, α4, and δ mRNA GABA-A in the frontopolar cortex in depressed suicide victims [82]. Suicide victims had decreased GABA-A α1 receptor subunit expression in the frontal cortex [89]. Later studies have shown polymorphisms of the γ-2 subunit in suicide attempts, suggesting that the longest variant of the GABA-A receptor γ-2 subunit is associated with protection against suicide attempts [90]. In both suicidal adults and adolescents, decreased expression of GABA-A receptors on platelets was also found [91].

### 2.6. Cholesterol

Low cholesterol in plasma is associated with reduced lipid rafts in the central nervous system and subsequent reduced synaptic plasticity and decreased serotonin activity that could predispose to aggression [67,92,93,94]. Some studies have implied that lower cholesterol levels could be a useful biological marker in depression [95]. Interest in cholesterol as a biomarker for suicide has been growing since the early 1990s, when a meta-analysis of randomised primary prevention trials of statins (cholesterol-lowering drugs) indicated that they reduced the risk of death by coronary events but increased the risk of suicide [96,97]. On the one hand, a recent meta-analysis of 65 studies (comprising up to 500,000 participants) showed that suicidal patients had lower total cholesterol levels than non-suicidal controls, associated with a 112% higher risk of suicidality [96]. On the other hand, Molero et al. (2020) found in a cohort of 1,149,384 participants that statins were not associated with suicidality [98]. Limited evidence suggests lower brain levels of cholesterol in suicide attempters [99].

## 3. Epigenetic Changes

Epigenetic mechanisms can produce heritable phenotypic changes without a modification in DNA sequence. They include DNA methylation, histone modification (methylation or acethylation), and microRNA (miRNA) [100]. Epigenetic regulation of BDNF, TrkB, HPA axis components, and GABA-A receptors plays an important role in suicidal behaviour [74,75,101]. In suicide completers, increased expression of DNA methyltransferase (DNMT), the enzyme that methylates DNA in the frontal cortex, as well as total DNA hypermethylation in the Wernicki cortex and prefrontal cortex have been found [74,75,79,102]. Enhanced total methylation of the DNA in the blood has also been observed [103]. Higher methylation levels of the BDNF, GABA-A receptor subunits, and TrkB receptor promoters, as well as the NR3C1 gene (coding glucocorticoid receptor promoter), have been observed in suicide victims than in controls [67,74,101]. The low TrkB expression in suicide victims is probably due to multiple epigenetic consequences, such as hypermethylation of its promoter and higher histone methylation (H3K27) [75,79]. Lower BDNF protein levels in suicides in the prefrontal cortex and hippocampus are probably due to a decrease in histone acetylation [75].

Post-mortem brain studies suggest that miRNAs may be involved in suicide [104,105]. MiRNAs are non-coding, endogenous, short chain RNAs that inhibit mRNA translation into proteins and may regulate neuroplasticity associated with BDNF or CREB (cMP response element-binding) [105]. Some researchers have revealed the upregulation of miR-19a-3p in the peripheral blood mononuclear cells of depressed patients with suicidal ideation [23]. The prefrontal cortex showed significant upregulation of miR-124, miR-139, miR-185, and miR-195, while miR-494 and miR-335 were downregulated in patients with suicide [23,106].

In summary, the epigenetic changes (especially methylation) of BDNF, HPA axis components, and GABA-A receptors play an important role in suicidal behaviour by regulating those proteins’ expressions.

## 4. Activity of Brain Structures in Suicidal Patients 

The frontal and prefrontal cortex plays an essential role in suicidal behaviour via its involvement in cognition, stress response, and suppression of impulsiveness [76,107,108]. Patients with a history of suicide attempts have altered prefrontal areas’ activation patterns associated with impaired decision making, risk reward, and social assessment [47]. The anterior cingulate cortex responsible for negative self-thinking and processing of emotional stimuli is strongly implicated in suicidality [109]. Relative to depressed non-attempters, suicide attempters showed greater activation of the anterior cingulated cortex when viewing emotionally expressive faces, indicating different processing of emotional stimuli [47]. Studies using fMRI have confirmed the role of ventral and dorsal prefrontal cortex (PFC) dysfunction in suicide pathophysiology in response to emotional and hedonic valence stimuli [8]. Structural MRI studies have shown lower grey matter volume and neural density in the ventral PFC and reduced hippocampal volumes in adults who had attempted suicide than in psychiatric controls [8,30].

Moreover, lower orbitofrontal activation during risky and safe choices is consistent with altered decision making [47]. In addition, the Nurses Global Assessment assessed abnormal resting-state functional connectivity in the frontolimbic system in patients with a higher risk of suicidality for suicidal risk with bipolar disorder [110].

## 5. Therapeutic Options to Prevent Suicide

Psychological treatments, pharmacotherapy (lithium, ketamine, esketamine, and clozapine), or electroconvulsive treatment reduce suicidal thoughts and behaviours [111,112,113]. There is no established effectiveness of drugs in preventing suicide [114,115]. However, drugs that exert evident anti-suicidal action exert joint anti-inflammatory action and increase the level of BDNF.

Some evidence suggests that psychological treatments (especially cognitive behavioural therapy) effectively reduce suicidal thoughts and behaviours, but their effect is seen to be deferred [45,116]. It is beneficial in the pediatric population [111]. Psychological interventions focus on strengthening skills in interpersonal communication, stress tolerance, and emotion regulation [45,117].

Electroconvulsive therapy (ECT) is safe and effective in reducing suicidal ideation in drug-resistant depression and schizophrenia in adults and adolescents, including population of pregnant women [118,119,120]. Interestingly, some data suggest that pre-ECT high level of TNF-α predicts a good response to ECT [121], and electroconvulsive therapy decreases inflammatory mediators [122].

### 5.1. Lithium

Lithium is a mood-stabilising drug with well-documented efficiency of anti-suicidal effects in drug-resistant depression and bipolar disorder [123,124,125,126]. A meta-analysis of 48 controlled trials (6674 participants) published by Ciprani et al. (2013) [127] found that lithium was more effective than a placebo in reducing the suicide rate in patients with depression. Long-term treatment with lithium reduced suicide attempts and deaths by approximately 20–60% in patients with depressive or bipolar disorders [124,126,128]. Lithium has been shown to decrease impulsivity, aggression, and cognitive decline in patients [26,124,129]; its action is probably connected to the neuroprotective effect on structures involved in emotional regulation, such as the prefrontal cortex. Lithium increases BDNF levels and reduces apoptotic processes [26,124,129,130]. Some studies have demonstrated that lithium inhibited microglial activation and exerted anti-inflammatory activity by inhibiting glycogen synthase kinase-3 (GSK3) [53,131]. It has been shown that inhibition of GSK-3β upregulated anti-inflammatory IL-10 production and reduced proinflammatory cytokines such as IL-1β, IL-6, TNF, IL-12, and IFN-γ activity [53,131,132]. A significant decrease in proinflammatory cytokines and attenuation of cyclooxygenase type 2 (COX-2) expression were evident after three months of lithium therapy [53,131,133]. The use of lithium to prevent suicide in depressed patients is limited due to its low therapeutic index and high toxicity [124]. During lithium therapy, adverse effects may occur which include thyroid insufficiency, kidney dysfunction, cardiac arrhythmia, neurologic symptoms, neurotoxicity delirium, and convulsions [134].

### 5.2. Clozapine 

Clozapine is a second-line, atypical antipsychotic approved for drug-resistant schizophrenia that effectively reduces suicidality and aggression/impulsivity [6,135]. Despite clozapine toxicity, especially the risk of agranulocytosis, a meta-analysis showed that continuous clozapine treatment in schizophrenia was associated with a significantly lower long-term all-cause mortality rate compared to other antipsychotics [136]. Moreover, some observations suggest that the anti-suicidal effect of clozapine is present not only in schizophrenia but also in bipolar disorder and severe forms of borderline personality disorder [128]. In preclinical models, it was found that chronic administration of clozapine exerted an anti-inflammatory effect [137,138].

### 5.3. Ketamine/Esketamine

Ketamine is a non-selective NMDA receptor antagonist used as an anaesthetic drug. Ketamine is a racemic mixture of S and R stereoisomers. Esketamine has a higher affinity for NMDA binding sites than the R isomer [6,139]. Ketamine and esketamine are the first pharmacological antidepressants with rapid and prolonged (3–14 days) action and an anti-suicidal effect in unipolar and bipolar depression [6,67,139]. The U.S. Food and Drug Administration (FDA) and European Medical Agency (EMA) registered intranasal esketamine in treating resistant depression with suicidal behaviour. Although ketamine is considered to be relatively safe, several side effects have been reported, such as agitation, dissociation, perceptual disturbance, abnormal sensations, increased blood pressure and increased heart rate, headache, and dizziness [140,141]. As compared with ECT, the ketamine is characterised by less cognitive impairment [142].

It has been found that ketamine reduced suicidal ideation in patients with depression and anxiety disorders [112,143]. The probable mechanism of action of ketamine is connected to the blockade of NMDA receptors and an increase in synaptic plasticity via the mTOR pathway and BDNF release [48,139,140]. Moreover, ketamine affects the kynurenine pathway and inhibits proinflammatory cytokine exacerbation [144,145]. Ketamine counteracts the increase in QUIN production induced by lipopolysaccharides (LPS) [139]. Ketamine has well-described effects on increasing total sleep and slow-wave sleep and reducing early-night awakening, which may be associated with ketamine anti-suicidal effects [144]. Ketamine also significantly impacts circadian rhythm systems via clock genes [144].

## 6. Drugs That Increase the Risk of Suicidality

Although antidepressants have a vital role in treating mood disorders, they present no evident anti-suicidal effect as compared with lithium, clozapine, and ketamine/esketamine [1,146,147]. Moreover, in rare cases, they could induce or exacerbate suicidal tendencies, during the first weeks of treatment, especially in children and adolescents [1,148]. Some of selective serotonin reuptake inhibitors (SSRI) may increase suicidal behaviour by stimulating depressed patients to act with preexisting suicidal thoughts [149]. In 2004, the FDA issued a black box warning that using antidepressants was associated with an increased risk of suicidal ideation and behaviour in people under 18. In 2007, the notification was expanded to include young adults under 25. Similarly, the EMA scientific committee concluded, in 2005, that suicide-related behaviours and aggression were more frequently observed in clinical trials among children and adolescents treated with SSRIs than those treated with placebos.

Several case reports have described suicidal ideation in previously psychiatrically healthy individuals after treatment with interferon-β [50].

In 2008, the FDA published a meta-analysis of 199 placebo-controlled trials of 11 anticonvulsant drugs. The FDA found that patients taking anticonvulsant drugs, such as gabapentin, tiagabine, and oxcarbazepine, had approximately twice the risk of suicidal behaviour or ideation as compared with patients receiving a placebo [150]. Epileptic patients appear to be at risk of developing treatment-induced psychiatric adverse effects [151].

## 7. Substance Misuse

Substance misuse is associated with an increased risk for suicide and suicide death [152,153,154]. Over 70% of adolescent suicides may be linked to drug and alcohol abuse and dependence [155]. The highest risk is associated with multiple dependence on alcohol, drug, and tobacco [154]. Studies have demonstrated that substance-dependent patients had impulsive personality or coexisting psychiatric disorders, especially mood disorders [156,157]. Among addictive substances, the most implicated in suicide is alcohol. Clinical evidence implies that alcohol is the second cause of suicide after depression [10]. Acute and chronic alcohol consumption may both propel suicide attempts through various mechanisms, i.e., increasing dysphoria, aggressiveness, and impulsivity; constricting cognition; and impairing generation and implementation of alternative coping strategies [10]. People under the influence of alcohol choose more radical and effective methods of dying by suicide, for example, throwing themselves under a moving vehicle [158]. Moreover, chronic alcohol use decreases serotonergic neurotransmission, which may also be associated with suicide behaviour [66]. However, this risk is modulated by various factors, including sociodemographic, clinical, treatment-related, and life situational characteristics [159]. Adequate dependence treatment may decrease the risk of suicide [152]. Data suggest that other dependences, especially when they coexist, are associated with an increased risk of suicidal behaviour. For example, it has been found that frequent cannabis use was associated with increased incidence of suicidal ideation, plans, or attempts [160,161].

## 8. Conclusions

Suicidal behaviour is associated with multiple risk factors such as psychiatric disorders (mainly depression), personality traits (aggression, impulsivity, and pessimism), and stressful life events. Suicide scales allow for quick screening but provide insufficient predictive validity. Therefore, it is worth searching for potential biological markers. Our current knowledge about the neurobiology of suicide is still limited. The core element of suicide etiopathogenesis seems to be neuroinflammation that subsequently stimulates the kynurenine pathway and causes serotonin depletion, and increases the level of quinolinic acid (NMDA receptor agonist). These processes lead to glutamatergic overactivation and decrease the production of BDNF that worsen neuroplasticity. Suicidal behaviour is also associated with overactivity of the HPA axis, which can cause a sequence of impairments, including stress control or cognitive dysfunction. Epigenetic mechanisms control the above-described neurobiological changes associated with suicidal behaviour. Patients with a history of suicide have altered activation patterns of prefrontal areas and consequently impaired decision making, risk reward, and social assessment.

Fortunately, suicide risk could be attenuated by appropriate psychological treatment, electroconvulsive treatment, and drugs (lithium, ketamine, esketamine, clozapine). Lithium, ketamine, and esketamine have anti-suicidal effects in patients with unipolar or bipolar depression, while clozapine exerts such an effect in patients with schizophrenia. Their mechanisms of action are different, but their common mechanisms are anti-inflammatory and neuroprotective, confirming the critical role of neuroinflammation in suicide risk.

## Figures and Tables

**Table 1:** Risk factors of suicide and matching treatment that decreases the risk of suicide.

Risk Factors of Suicide	Matching Treatment That Decreases the Risk of Suicide
Emotional traits: aggression, impulsivity, pessimism	Lithium (in depression and bipolar disorder, delayed effect), clozapine (schizophrenia)
Early life stress	-
Depression	Ketamine/esketamine (in depression, rapid effect), lithium (in depression and bipolar disorder, delayed effect), electroconvulsive therapy, psychotherapy, transcranial magnetic stimulation
Schizophrenia	Clozapine
Other psychiatric disorders	Psychotherapy
Alcohol dependence and other dependence	Treatment of alcohol or substance abuse

**Table 2:** The selected potential markers of suicidal behaviour in prefrontal cortex, hippocampus, and peripheral tissue (blood or cerebrospinal fluid).

	Prefrontal Cortex	Hippocampus	Peripheral Tissue
5-HIAA	-	↑	CSF ↓ platelets ↓
Serotonin transporter	↓	-	↓ platelets
GABA-A receptor	Contradictory information	↑	↓
CRH	↑	↑	↑
CRH receptor type 1	↓		
Cortisol	No data	No data	↑ plasma, CSF
BDNF	↓	↓	↓ serum
IL-1	↑	No data	↑ blond
IL-6	↑	No data	↑ blood, CSF
IL-8	-	No data	↓ blood, CSF
Quinolinic acid	↑	No data	CSF, blood ↑
Cholesterol	Decrease only in violence	-	↓
DNA hypermethylation	↑	↑	↑
miR-124, miR-139, miR-185, miR-195	↑	No data	No data
miR-494, miR-335	↓	No data	No data
miR-19a3p	↑	No data	Blood mononuclear cells ↑

5-HIAA—5-hydroxyindoloacetic acid; 5HT—serotonin; GABA-A receptor—γ-aminobutyric acid receptor; CRH—corticotrophin releasing hormone; BDNF—brain-derived neurotrophic factor; IL—interleukin; CSF—cerebrospinal fluid, no changes.

## Data Availability

No new data were created or analysed in this study.
